# Distribution and Prevalence of Plant-Parasitic Nematodes of Turfgrass at Golf Courses in China

**DOI:** 10.3390/biology11091322

**Published:** 2022-09-06

**Authors:** Yinglu Dong, Peiyuan Jin, Huangwei Zhang, Jian Hu, Kurt Lamour, Zhimin Yang

**Affiliations:** 1 College of Agro-Grassland Science, Nanjing Agricultural University, Nanjing 210095, China; 2 Department of Entomology and Plant Pathology, University of Tennessee, Knoxville, TN 37996, USA

**Keywords:** plant-parasitic nematode, turfgrass, golf course, distribution, prevalence

## Abstract

**Simple Summary:**

Plant-parasitic nematodes (PPNs) are one of the most important pests to cause infectious diseases on turfgrass. Numerous studies have been conducted on PPNs associated with turfgrass worldwide. However, little published research is available on PPNs associated with turfgrass in China. Information on the occurrence, distribution, diversity, and dominant species of PPNs associated with turfgrass in different regions of China is limited, making it challenging to design suitable management practices for control. In this study, we assessed morphological characteristics and utilized molecular methods to identify PPNs from turfgrass soil samples. The purpose of this study was to determine the species, distribution, incidence, and diversity of PPNs associated with turfgrass at golf courses in North, East, and South China (NC, EC, and SC, respectively). The results provide valuable information for the management of PPNs on turfgrass in China.

**Abstract:**

We sampled 127 turfgrass soil samples from 33 golf courses in NC, EC, and SC for plant-parasitic nematodes (PPNs). PPNs were extracted from soil samples using the shallow dish method and were identified at the genus or species levels with a combination of morphological and molecular methods. The results revealed 41 species of nematode belonging to 20 genera and 10 families. Nine genera are new records of PPNs associated with turfgrass in China. The PPNs show strong geographical distributions. Of the 20 genera, *Helicotylenchus*, *Paratrichodorus*, *Hoplolaimus*, *Meloidogyne*, *Hemicriconemoides*, and *Mesocriconema* showed higher infestation and frequency, and most of these genera had numbers in soil samples above established damage thresholds. Four golf courses had soil samples with PPNs > 30%, indicating the potential for nematode damage. The biodiversity indices *H’*, *SR*, *J’*, *λ*, and *H_2_* showed significant differences among different regions and turfgrass species; *H’*, *SR*, *J’*, and *H_2_* were significantly higher in EC than in NC and SC, while *λ* was lowest in EC. Creeping bentgrass had the highest *H’*, *SR*, *J’*, and *H_2_* and the lowest *λ* in comparison with seashore paspalum and hybrid bermudagrass. These findings provide baseline information on the occurrence of turfgrass-associated PPNs in China, and have important implications for the effective management of PPNs causing damage on turfgrass.

## 1. Introduction

Turfgrasses are widely planted in courtyard, urban, and athletic fields, serving important functions in beautifying the environment, preventing soil erosion, purifying the air, and providing safe surfaces for recreational activities [1]. However, the health of turfgrass is frequently threatened by diverse abiotic and biotic factors, especially at golf courses with high maintenance [2]. Plant-parasitic nematodes (PPNs) are one of the most important biotic factors causing turfgrass disease. They mainly damage the roots by limiting root development and increasing root susceptibility to other soilborne pathogens, such as fungi, bacteria, and viruses [3]. It is difficult to accurately diagnose problems caused by nematodes because the aboveground symptoms caused by nematodes are often delayed, diverse, and relatively nonspecific [4]. The aboveground turf may display wilting, chlorosis, or patchiness when the belowground roots are attacked by nematodes [5,6]. These symptoms are often misdiagnosed as caused by other pathogenic or opportunistic microorganisms. Numerous studies have been conducted on PPNs associated with turfgrass worldwide [7]. However, limited published research is available for PPNs associated with turfgrass in the southern region of China [8]. Additional studies are needed to gain a better understanding of nematode impacts on turfgrass in China, and can provide valuable information for managing high quality turfgrass.

The species of PPNs on turfgrass are diverse and different across regions and countries. Twenty-nine species of PPNs belonging to 22 genera in 15 families have been found to be associated with bermudagrass (*Cynodon dactylon*), creeping bentgrass (*Agrostis stolonifera*), and zoysiagrass (*Zoysia japonica*) in North Carolina and South Carolina [9]. *Helicotylenchus* sp., *Mesocriconema* sp., *Hoplolaimus* sp., *Tylenchorhynchus* sp., *Belonolaimus longicaudatus*, *Meloidogyne* sp., and *Paratrichodorus* sp. were the most prevalent and abundant species in the local golf courses [7]. In Belgium, over 50 species/taxa of PPNs belonging to 23 genera in 9 families have been recorded on turfgrass [10]. *Helicotylenchus pseudorobustus* and *Meloidogyne naasi* were the most prevalent nematodes. Twenty-eight species/taxa belonging to 16 genera in 12 families of PPNs were found at 13 golf courses in Korea [11], with *Helicotylenchus microlobus*, *Mesocriconema nebraskense*, *Tylenchorhynchus claytoni*, *Mesocriconema* sp., and *Meloidogyne graminicola* the most prevalent species in all management zones [11]. Twenty-three nematode genera are known to be associated with turfgrasses in Australia [12]. In China, a recent survey identified *Helicotylenchus* sp., *Mesocriconema* sp., *Meloidogyne* sp., *Hemicriconemoides* sp., *Xiphinema* sp., *Pratylenchus* sp., *Hoplolaimus* sp., *Paratrichodorus* sp., *Filenchus* sp., and *Ogma* sp. at golf courses in Guangdong [8]. The dominant species were *Mesocriconema xenoplax* and *Meloidogyne graminis*. *Helicotylenchus dihystera*, *Mesocriconema xenoplax*, *Hoplolaimus* sp., *Paratrichodorus* sp., *Filenchus* sp., and *Ogma* sp. in this new record of PPNs associated with turfgrasses in China [8].

Although there are diverse species of PPNs associated with turfgrasses [12], and multiple species are generally present at a particular location, this does not necessarily mean that infested turfgrass exhibits signs or symptoms of nematode damage [12]. Most nematodes only cause visible damage when the population is very high and the turfgrass is stressed. PPNs vary in their virulence, and different PPNs have different damage thresholds [13]. Nevertheless, nematodes consistently cause severe damage, and the most damaging PPNs on turfgrasses may vary across different regions and countries [6,13]. The sting nematode, *Belonolaimus longicaudatus*, is considered the most damaging PPN on bermudagrass in the southeast United States, while the root-knot nematodes (*Meloidogyne* spp.) pose a serious threat to turfgrasses in the southern United States [14,15]. The southern sting nematode (*Ibipora lolii*) is by far the most destructive nematode of turfgrasses in Australia [6]. The damage threshold for *B. longicaudatus* on turfgrasses is considered to be less than 25 nematodes/100 mL soil, and the damage threshold for *I. lolii* is likely to be similar to *B. longicaudatus* [16,17,18]. Until recently, information on the occurrence, distribution, diversity, and dominant species of PPNs associated with turfgrass in different regions of China has been limited, making it challenging to design suitable management practices for control.

In this study, we assessed morphological characteristics and utilized molecular methods to identify PPNs from turfgrass soil samples. The purpose of this study was to determine the species, distribution, incidence, and diversity of PPNs associated with turfgrass from golf courses in North, East, and South China (NC, EC, and SC, respectively). The results provide valuable information for the management of PPNs on turfgrass in China.

## 2. Materials and Methods

### 2.1. Sample Collection

**Figure 1:**
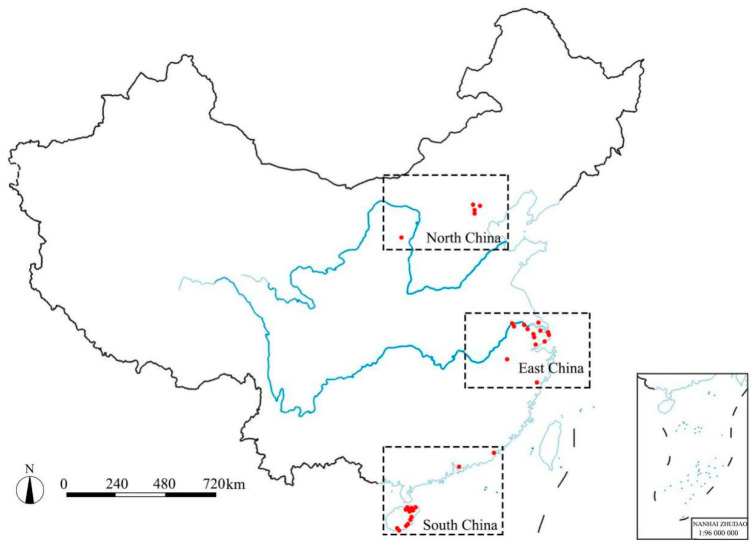
The distributions of the thirty-three sampled golf courses in North, East, and South China.

Numerous studies on turfgrass-associated PPNs have focused on turfgrass soil [6,7]. In order to effectively compare the results in the current study with those in previous studies, soil samples were collected from creeping bentgrass, Kentucky bluegrass (*Poa pratensis* L.), hybrid bermudagrass (*Cynodon dactylon* × *C. transvaalensis*), and seashore paspalum (*Paspalum vaginatum*) at 33 golf courses in NC, EC, and SC during the period from November 2020 to October 2021 (Table 1). The sampling time varied in different regions due to differing periods of time with extensive turfgrass damage in these regions. All sampled golf courses were constructed with sandy soil and were equipped with standard irrigation and drainage systems. Creeping bentgrass and Kentucky bluegrass turf were seeded, while hybrid bermudagrass and seashore paspalum turf were transplanted. There were 5, 14, and 14 golf courses sampled in NC, EC, and SC, respectively (Figure 1), and a total of 12, 52, and 63 soil samples were collected in NC, EC, and SC. Areas with symptoms of turfgrass damage (stunted growth, less dense turf stands, shoot chlorosis, root discolor) were preferred during sampling. Usually, 15–20 soil cores (5 cm in diameter and 15 cm in depth) were collected and merged into one composite soil sample per location (fairway or green). However, if multiple fairways or greens at one golf course showed different symptoms (based on the observation and experience of golf course superintendents), multiple composite samples per golf course were collected. Meanwhile, at least one symptomatic sample and one adjacent apparently healthy sample were collected from 18 golf courses in order to compare the relationship between health status and nematode density. Samples were placed in plastic bags, immediately transported back to the laboratory with ice bags, and stored at 4 °C before analysis to minimize changes in nematode populations [19]. Table 1 summarizes the detailed information on the collected samples. The disease symptoms, turfgrass species, sampling date, and management zone were recorded. In total, one hundred and twenty-seven samples were collected and processed for nematode identification.

### 2.2. Nematode Extraction and Identification

Samples consisting of multiple cores of turf were gently mixed to break the cores before extraction; 100 cm^3^ of soil mixture for each sample was used for nematode extraction. Nematodes were extracted from soil samples using the shallow dish method [20,21].

Nematodes were identified to the genus level, and to the species level if possible, based on morphological characteristics; adult females of PPN species were hand-picked and processed to glycerin for identification. The data were recorded as the number of nematodes per 100 cm^3^ of soil. Nematodes were counted under a stereomicroscope (OLYMPUS DP80) in both larval and adult stages, and morphological identification was carried out under an optical microscope (OLYMPUS DP80). Individual nematodes (mainly mature females) representing each genus or species were picked, killed by heating, and fixed as temporary slide specimens for observation [9]. Nematodes were identified according to the classification system of Goodey (1963) [22] and the books *Plant* *Nematology* and *Taxonomy of Plant Nematodes* [23,24]. The body length, head, lip area, stylet, medium bulb, tail, reproductive system, and digestive system of nematodes were the key points used for morphological identification [25].

Molecular identification was a complementary option when it was difficult to identify nematodes to the genus or species level by morphological characteristics. Meanwhile, in order to confirm the accuracy of morphological identification, all nematodes showing morphological differences were further identified by the molecular method. DNA was extracted by the freeze–thaw method [26]; 28S LSU D2-D3 partial fragment sequence was amplified with the forward primer D2A (5′-ACAAGTACCGTGAGGGAAAGTTG-3′) and the reverse primer D3B (5′-TCGGAAGGAACCAGCTACTA-3′) [27], while 18S SSU partial fragment sequence was amplified with the forward primer 18S965 (5′-GGCGATCAGATACCGCCCTAGTT-3′) and the reverse primer 18S1573R (5′-TACAAAGGGCAGGGACGTAAT-3′) [28]. PCR amplification was conducted in a final volume of 25 μL: 1 μL of template DNA, 2 μL of each primer (10 μM), 12.5 μL of 2 × Taq Master Mix (Tsingke Biotech Co. Ltd., Nanjing, China), and 7.5 μL ddH_2_O. Amplification was performed with the following parameters: initial denaturation at 94 °C for 4 min followed by 37 cycles of denaturation at 94 °C for 30 s, annealing at 56 °C for 40 s, extension at 72 °C for 1 min, and a final elongation step at 72 °C for 10 min. PCR products were purified and sent to Tsingke Biotechnology Co., Ltd. for sequencing. Each sequence was blasted using the Basic Local Alignment Search Tool (blastn) at NCBI. Nematodes were identified to the genus or species levels with the combinations of morphological and molecular methods described above.

### 2.3. Data Analysis

The main nematode genera in each region were selected according to the criteria of abundance greater than 1. The dominance of PPNs was expressed as the percentage of the number of PPNs in the total number of nematodes. The prevalence of PPNs was analyzed based on infestation, frequency, abundance, max individuals per 100 cm^3^ of soil, and percentage of samples above damage threshold. Golf course infestation was defined as the number of courses having a particular nematode species divided by the total number of courses in this region, and was expressed as a percentage. Frequency and abundance were calculated according to Sawadogo and Thio (2009) [29]:

Frequency (%) = *P*/*T* × 100

Abundance = *lg* [(*n_i_*/*P*) + 1]

*T* = total number of samples

*P* = number of positive samples

*n_i_* = number of nematodes in genus *i*

At the same time, the damage threshold was defined as the number of nematodes required to cause economic damage on a given turfgrass species based on findings from Nelson (1995) [30] and Stirling (2021) [6]. Several diversity indices were calculated following Yeates and Bird (1994) [31]:

$$\mathrm{Shannon}–\mathrm{Weaver}\;\mathrm{diversity}\;H^{\prime }=-\sum_{i=1}^{s}pi\ln pi$$

Richness *SR* = (*s* − 1) *ln N*

Evenness *J′* = *H′*/*H′ _max_*

$${\mathrm{Simpson}}^{\prime }s\;\mathrm{dominance}\;\lambda =\sum_{i=1}^{s}p_{i}^{2}$$

$$\mathrm{Diversity}\;H_{2}=-\ln\lambda$$

*s* = number of taxa (species) in the sample

*N* = total number of nematodes identified in the sample

pi=proportion of individuals of genus i in the total population

*H*′ *_max_* = *ln s*

The Shannon–Weaver diversity (*H′*) is commonly used to assess diversity; however, as it may be dominated by the most abundant taxa or the overall number of taxa, both evenness and richness were calculated as well. Simpson’s dominance measure (*λ*) is used to assess dominance, and its log_e_ transformation offers an alternative measure of diversity (*H*_2_) [31].

Differences in the diversity indices were analyzed by Duncan’s multiple range test (*p* < 0.05) in IBM SPSS Statistics Version 23.0 (IBM Corporation, Armonk, NY, USA).

## 3. Results

### 3.1. Classification and Distribution of Plant-Parasitic Nematodes

Forty-one species of PPNs in twenty genera of ten families from four orders were identified in the 127 soil samples collected from 33 golf courses in NC, EC, and SC. Nematodes in the order Tylenchida accounted for the largest proportion, including the genii *Helicotylenchus*, *Hoplolaimus*, *Rotylenchulus*, *Filenchus*, *Discotylenchus*, *Ecphyadophora*, *Labrys*, *Criconemoides*, *Mesocriconema*, *Hemicycliophora*, *Hemicriconemoides*, *Tylenchorhynchus*, *Hirschmanniella*, *Ditylenchus*, *Meloidogyne* and *Heterodera*. The remaining three orders (Triplonchida, Dorylaimida, Aphelenchida) contained four genera (*Paratrichodorus*, *Xiphinema*, *Paralongidorus*, *Aphelenchoides*). Detailed information on the classification of PPNs is shown in Table 2.

Of the 41 species of PPNs, only one species (*Filenchus* sp.) was found in all three regions (NC, EC, and SC), appearing at ten golf courses, while thirteen species were found in two regions. A total of seven, thirteen, and seven species of PPNs were found exclusively in NC, EC, or SC, respectively. *Ditylenchus dipsaci*, *Mesocriconema* sp., *Hemicriconemoides* sp., *Meloidogyne graminis*, *Helicotylenchus* sp., *Helicotylenchus microlobus*, *Helicotylenchus dihystera*, *Filenchus* sp., *Paratrichodorus minor*, and *Xiphinema ifacolum* were found to be associated with at least two turfgrass species, while the rest species of PPNs were exclusively found to be associated with specific turfgrass species (Table 2).

### 3.2. Prevalence of the Main PPNs

Table 3 shows the specific information on the infestation, frequency, abundance, and damage threshold of the main PPN genera (abundance > 1) at golf courses. There were two, six, and five genera of PPNs with abundance > 1 in NC, EC, and SC, respectively. *Helicotylenchus* species were found in all sampling regions (the data in EC are not shown in the table because the abundance was less than 1), with high infestation, frequency, and abundance in NC and SC; 33.33% of the samples from SC had *Helicotylenchus* above the damage threshold. In EC, *Paratrichodorus* had the highest infestation (64.29%) and the highest frequency (59.62%); the proportion of soil samples with *Paratrichodorus* above the damage threshold accounted for 9.68%. *Xiphinema* had the second highest infestation rate in EC, with an infestation, frequency, and abundance of 42.86%, 23.08%, and 1.86, respectively, and the number of samples above the damage threshold accounted for 8.33%. *Meloidogyne* had a low infestation rate (21.43% in EC and 16.67% in SC), with high abundance (1.61 in EC and 1.37 in SC). The proportion of samples above the damage threshold was 28.57% in EC and 12.5% in SC.

### 3.3. PPNs and Soil Health

This research revealed that the density of PPNs in turfgrass soil is directly reflected in soil health; the dominance of PPNs in healthy soil was generally lower than 30% [32,33]. In this study, the density and dominance of PPNs in healthy and problematic samples were compared (Table 4) in order to better identify the potential issues caused by PPNs on turfgrass. Of the eighteen golf courses investigated, four revealed dominance of PPNs higher than 30%, while the density and dominance of problematic samples in three golf courses (Nanjing Sun Island, Shenzhen Sand River, and Shanghai Tomson) were higher than those of healthy samples. In samples from Shanqin Bay golf course, the dominance of PPNs in healthy and problematic samples was 61.70% and 50.12%, respectively, significantly higher than 30%. Meanwhile, the density of PPNs in healthy samples was significantly higher than that in problematic samples from this golf course. Of the other fourteen golf courses with dominance of PPNs lower than 30%, the density and dominance of PPNs in healthy samples in Nanjing Zhongshan (H: 24.69%, P: 15.31%), Shanghai Links (H: 10.83%, P: 3.86%), Anhui Huangshan Pine (H: 9.33%, P: 4.18%), and Shenzhou Peninsula (H: 14.86%, P: 3.50%) golf courses were higher than those in problematic samples, while the other golf courses had similar dominance of PPNs in both healthy and problematic samples; the density of PPNs was consistently higher in problematic samples at these golf courses.

### 3.4. Nematode Diversity

The indices *H’*, *SR*, *J’*, *λ*, and *H_2_*, which reflect the biodiversity of PPNs, showed significant differences at golf courses in NC, EC, and SC (Table 5). *H’*, *SR*, *J’*, and *H_2_* were significantly higher in EC than in NC and SC, while *λ* was lowest in EC. *H’*, *J’*, and *H_2_* were lowest and *λ* was highest in SC. The impact of turfgrass species on the biodiversity indices of PPNs are presented in Table 6. *H’*, *SR*, *J’*, *λ*, and *H_2_* showed significant differences among the three species of turfgrass. *H’*, *SR*, *J’*, and *H_2_* were significantly higher in creeping bentgrass than in seashore paspalum and hybrid bermudagrass, while *λ* was lowest in creeping bentgrass. *H’*, *J’*, and *H_2_* were lowest and *λ* was highest in seashore paspalum.

## 4. Discussion

There is a lack of systematic research on PPNs on turfgrass at golf courses in China, as previous studies have often been limited to certain regions [8,34,35]. This study expanded the scope of investigations to a country-wide scale. In total, 41 species of PPNs from 20 genera were identified in 127 soil samples collected from 33 golf courses in NC, EC, and SC. It has previously been reported that a total of 15 nematode genera are associated with turfgrass in Guangdong, China [8], and 73% of these were documented in the current study as well. *Ditylenchus, Hemicycliophora, Heterodera, Rotylenchulus, Hirschmanniella, Discotylenchus, Ecphyadophora, Labrys*, and *Paralongidorus* were the newly recorded nematode genera on turfgrass in China. These results reveal a high diversity of PPNs associated with turfgrasses at golf courses in China.

Of the 41 species identified in this study, only one species (*Filenchus* spp.) was detected in all three regions (NC, EC, and SC), with most species exhibiting strong regional distributions. For example, *Tylenchorhynchus* *annulatus*, *Criconemoides* *annulatus*, and *Heterodera* *avenae* were only found in NC, *Ditylenchus destructor* and *Meloidogyne incognita* were only found in EC, and *Helicotylenchus indicus* and *Aphelenchoides pseudogoodeyi* were only found in SC. Similar regional distributions of turfgrass associated-PPNs have been observed in other countries [10,29,34,35,36,37,38,39].

Previous studies have shown that the nematode genera Belonolaimus, Mesocriconema, Helicotylenchus, Hemicycliophora, Hoplolaimus, Paratrichodorus, Pratylenchus, Meloidogyne, Trichodorus, Tylenchorhynchus, and Xiphinema are associated with turfgrasses [40,41,42]. Similar results were revealed in this study as well. Table 3 shows the nematode genera with abundance greater than 1, which can potentially cause damage to turfgrass. The turfgrass-associated genera Helicotylenchus, Paratrichodorus, Hoplolaimus, Meloidogyne, Hemicriconemoides, and Mesocriconema showed high infestation and frequency in this study, and most of these genera had numbers in soil samples above the damage threshold. For example, Helicotylenchus was widely distributed with a high infestation rate in NC and SC, and the number of Helicotylenchus in a soil sample from Shanqin Bay golf course significantly exceeded the damage threshold [13]. Meloidogyne has been reported to cause great economic losses and severely damage the root system of turfgrass [43,44], and was found to exceed the damage threshold in soil samples from Shenzhen Sand River golf course [13]. In this study, we found that more genera appeared to cause damage on turfgrass in EC and SC compared to NC, and the number of nematode genera (abundance > 1) did not exceed the damage threshold in NC. This is reasonable, as the EC and SC regions have increased rainfall, soil moisture, and humidity profiles, and several nematode genera showed a clear trend of increasing prevalence in humid regions compared to dry regions [17,29]. These results suggest that close attention should be paid to those nematode genera with high abundance in order to prevent severe damage to turfgrass in China, especially in EC and SC.

Beginning in the late 1980s, nematodes have been used as an indicator of overall soil health [45]. In this study, we compared the density and dominance of PPNs in healthy and problematic soil samples in order to better understand potential problems caused by nematodes. However, for most of the investigated golf courses the soil samples had PPNs with dominance less than 30%, which indicated that the problematic soils and turfgrasses from these golf courses were unlikely to be caused by PPNs. Only four golf courses had soil samples in which dominance of PPNs was more than 30%, and three of them showed density and dominance of PPNs that were higher in problematic soil than in healthy soil. Moreover, four golf courses (Nanjing Sun Island, Shenzhen Sand River, Shanghai Tomson, and Wanning Shanqin Bay) were documented as having nematode problems according to communications with their respective golf course superintendents, which indicates that the density and dominance indices are valuable for nematode problem diagnosis. At present, definitive diagnosis of problems caused by nematodes is challenging because the symptoms of nematode damage are relatively nonspecific [5,6]. Monitoring the density and dominance of PPNs in healthy and problematic turfgrass soil can help to better ascertain the impact of nematodes on total plant health.

*H’*, *SR*, *J’*, *λ*, and *H_2_* are important indices for describing biodiversity and analyzing the impact of different factors on the species distribution of PPNs [7,46]. In our study, all the indices were significantly different among different regions and turfgrass species, similar to the results observed in previous studies [7]. This reveals the effect of different habitats (including turfgrass species and geographical regions) on the diversity of nematode species in turfgrass ecosystems. In this study, soil samples from golf courses in SC and seashore paspalum had the lowest *H’*, *J’*, and *H_2_* values, indicating a lower diversity of species and a potentially higher risk of nematode issues. Seashore paspalum, a warm season turfgrass, is widely planted in SC and is reported to be more susceptible to sources of biotic stress (e.g., pathogenic fungi) than other turfgrass species, which is consistent with the findings in this study [47]. Further investigation is needed in order to monitor the progress of nematode impacts on seashore paspalum.

During our survey of PPNs on turfgrass, we found that the soil samples from Jinji Lake and Suzhou Taihu golf course contained higher proportions of predatory nematodes, while PPNs were often found in lower proportions. This finding suggests that the presence of predatory nematodes may influence the propagation of PPNs. Predatory nematodes have been suggested as potential biocontrol materials for nematode diseases in a few previous studies [48,49]. However, very little confirmed evidence has shown that they play a vital role in mitigating the damage caused by PPNs. While the roles of predatory nematodes (including as bacterivores, fungivores, and omnivores) need to be further investigated, they may help better control problems caused by PPNs and improve overall soil health [50].

## 5. Conclusions

In conclusion, a total of 41 species of PPNs belonging to 20 genera and 10 families were found to be associated with different species of turfgrasses at golf courses in NC, EC, and SC; of these, nine genera are new records of PPNs associated with turfgrass in China. This study revealed the distribution and prevalence of PPNs on turfgrass at golf courses in China, highlighting the most prevalent genera associated with lowered soil health and turf damage. The results suggest that comparing the density and dominance indices in healthy and problematic soil samples may help to accurately assess the deleterious impacts caused by PPNs. The biodiversity indices of PPNs were significantly different among different regions and turfgrass species, indicating differential risk of nematode issues in these regions or on different species of turfgrass. This study can provide baseline information on the occurrence of turfgrass-associated PPNs in China, and the results have important implications for the management of PPNs on turfgrass in China. However, certain the indices, especially density and dominance, need to be interpreted carefully, as they are based on single samples and lack reproducibility. Further research is needed, and sampling throughout the season should be considered, along with increased sampling at single golf courses, allowing much stronger conclusions to be drawn.

## Figures and Tables

**Table 1:** Detailed information on turfgrass soil samples collected from different regions of China.

Region	Sampling Date	Golf Course	Turfgrass Species	No. of Samples
NC	July 2021	Beijing Qinghewan	Creeping bentgrass	2
	July 2021	Beijing Laffitte	Creeping bentgrass	2
	August 2021	Beijing Yuyang	Creeping bentgrass	4
	October 2021	Beijing CBD	Creeping bentgrass	2
	October 2021	Shaanxi Yulin Desert	Kentucky bluegrass	2
EC	May 2021	Nantong Xiao Yang Kou	Creeping bentgrass, Seashore paspalum	2
	July 2021	Nanjing Sun Island	Creeping bentgrass	6
	August 2021	Nanjing Zhongshan	Creeping bentgrass	2
	July 2021	Suzhou Taihu	Creeping bentgrass	8
	July 2021	Taicang East Sea	Creeping bentgrass	2
	July 2021	Suzhou Jinji Lake	Creeping bentgrass	6
	September 2021	Changzhou Kingswan	Creeping bentgrass	5
	September 2021	Suzhou Shuangshan	Creeping bentgrass	4
	May 2021	Huzhou Hotspring	Creeping bentgrass	3
	July 2021	Haining Lake Hill	Creeping bentgrass	2
	July 2021	Shanghai Sino-Bay	Creeping bentgrass	4
	August 2021	Shanghai Links	Creeping bentgrass	2
	August 2021	Shanghai Tomson	Creeping bentgrass	2
	September 2021	Huangshan Pine	Hybrid bermudagrass	4
SC	November 2020	Hainan Meilan	Hybrid bermudagrass	4
	November 2020	Hainan Meilangwan	Seashore paspalum	5
	November 2020	Hainan West Coast	Seashore paspalum	6
	November 2020	Hainan Meishi	Hybrid bermudagrass	4
	November 2020	Hainan Mission Hills	Hybrid bermudagrass	4
	November 2020	Haikou Three Miles	Hybrid bermudagrass	7
	November 2020	Boao Asia BBS	Hybrid bermudagrass, Seashore paspalum	4
	November 2020	Qionghai White Stone Hot Spring	Seashore paspalum	2
	July 2021	Shenzhou Peninsula	Seashore paspalum	4
	November 2020	Sanya Blue Bay	Seashore paspalum	6
	July 2021	Wanning Shanqin Bay	Seashore paspalum	4
	September 2021	Sanya Luhuitou	Hybrid bermudagrass	3
	September 2021	Guangdong Chaoshan	Creeping bentgrass	2
	October 2021	Shenzhen Sand River	Hybrid bermudagrass, Seashore paspalum	8

**Table 2:** The classification and distribution of plant-parasitic nematodes in North, South, and East China.

Order	Nematode Classification	Region	Golf Course	Turfgrass Species	No. of Courses
	Order Tylenchida, family Anguinidae				-
1	*Ditylenchus* spp.	EC	Nanjing Zhongshan	Creeping bentgrass	1
2	*Ditylenchus destructor*	EC	Suzhou Taihu	Creeping bentgrass	1
3	*Ditylenchus dipsaci*	EC	Huzhou Hotspring, Shanghai Sino-Bay, Shanghai Links	Creeping bentgrass	3
		SC	Wanning Shanqin Bay, Hainan Mission Hills	Hybrid bermudagrass, Seashore Paspalum	2
4	*Ditylenchus* cf. *microdens*	NC	Shaanxi Yulin Desert	Kentucky bluegrass	1
	Order Tylenchida, family Belonolaimidae				-
5	*Tylenchorhynchus annulatus*	NC	Beijing Laffitte	Creeping bentgrass	1
	Order Tylenchida, family Criconematidae				-
6	*Criconemoides annulatus*	NC	Beijing Laffitte	Creeping bentgrass	1
7	*Mesocriconema* spp.	NC	Shaanxi Yulin Desert	Kentucky bluegrass	1
		SC	Haikou Three Miles, Guangdong Chaoshan, Shenzhen Sand River	Creeping bentgrass, Seashore Paspalum, Hybrid bermudagrass	3
8	*Mesocriconema curvatum*	NC	Beijing Laffitte, Beijing Yuyang	Creeping bentgrass	2
9	*Hemicycliophora* *conida*	NC	Shaanxi Yulin Desert	Kentucky bluegrass	1
10	*Hemicriconemoides* spp.	EC	Nantong Xiao Yang Kou	Creeping bentgrass	1
		SC	Hainan West Coast, Wanning Shanqin Bay, Shenzhou Peninsula, Sanya Luhuitou, Haikou Meilangwan, Hainan Mission Hills	Seashore Paspalum, Hybrid bermudagrass	6
	Order Tylenchida, family Heteroderidae				-
11	*Heterodera avenae*	NC	Beijing CBD	Creeping bentgrass	1
12	*Meloidogyne graminis*	EC	Changzhou Kingswan, Nanjing Zhongshan	Creeping bentgrass	2
		SC	Shenzhen Sand River	Hybrid bermudagrass, Seashore Paspalum	1
13	*Meloidogyne incognita*	EC	Suzhou Jinji Lake	Creeping bentgrass	1
	Order Tylenchida, family Hoplolaimidae				-
14	*Helicotylenchus* spp.	EC	Suzhou Jinji Lake, Suzhou Shuangshan, Shanghai Sino-Bay, Huangshan Pine	Creeping bentgrass, Hybrid bermudagrass	4
		SC	Haikou Meishi, Haikou Meilangwan, Boao Asia BBS, Qionghai White Stone Hot Spring	Hybrid bermudagrass, Seashore Paspalum	4
15	*Helicotylenchus microlobus*	NC	Beijing Qinghewan, Beijing Laffitte	Creeping bentgrass	2
		SC	Hainan Mission Hills, Haikou Three Miles, Haikou Meilan, Wanning Shanqin Bay	Hybrid bermudagrass, Seashore Paspalum	4
16	*Helicotylenchus digitiformis*	EC	Nantong Xiao Yang Kou	Creeping bentgrass	1
17	*Helicotylenchus dihystera*	NC	Beijing Yuyang	Creeping bentgrass	1
		SC	Shenzhou Peninsula, Hainan West Coast, Guangdong Chaoshan	Hybrid bermudagrass, Seashore Paspalum	3
18	*Helicotylenchus paraplatyurus*	SC	Hainan Mission Hills	Hybrid bermudagrass	1
19	*Helicotylenchus pseudorobustus*	NC	Beijing CBD	Creeping bentgrass	1
		SC	Hainan West Coast	Seashore Paspalum	1
20	*Helicotylenchus rotundicauda*	EC	Nantong Xiao Yang Kou	Creeping bentgrass	1
21	*Helicotylenchus indicus*	SC	Shenzhen Sand River	Hybrid bermudagrass, Seashore Paspalum	1
22	*Hoplolaimus columbus*	EC	Suzhou Jinji Lake, Nanjing Zhongshan, Shanghai Sino-Bay, Links, Shanghai Tomson	Creeping bentgrass	5
		SC	Hainan West Coast, Guangdong Chaoshan	Seashore Paspalum, Creeping bentgrass	2
23	*Rotylenchulus* spp.	SC	Haikou Three Miles	Hybrid bermudagrass	1
	Order Tylenchida, family Pratylenchidae				-
24	*Hirschmanniella* *oryzae*	EC	Huzhou Hotspring	Creeping bentgrass	1
	Order Tylenchida, family Tylenchidae				-
25	*Filenchus* spp.	NC	Beijing Qinghewan, Beijing Yuyang	Creeping bentgrass	2
		EC	Suzhou Jinji Lake, Suzhou Shuangshan	Creeping bentgrass	2
		SC	Hainan Meilan, Haikou Meilangwan, Haikou Meishi, Hainan West Coast, Haikou Three Miles, Qionghai White Stone Hot Spring	Hybrid bermudagrass, Seashore Paspalum	6
26	*Filenchus discrepans*	EC	Suzhou Taihu, Taicang East Sea	Creeping bentgrass	2
27	*Filenchus misellus*	EC	Huangshan Pine	Hybrid bermudagrass	1
28	*Discotylenchus* spp.	NC	Beijing CBD	Creeping bentgrass	1
		EC	Shanghai Tomson	Creeping bentgrass	1
29	*Ecphyadophora quadralata*	EC	Suzhou Taihu	Creeping bentgrass	1
30	*Labrys* *khuzestanensis*	SC	Shenzhou Peninsula	Seashore Paspalum	1
31	*Labrys chinensis*	SC	Wanning Shanqin Bay	Seashore Paspalum	1
	Order Triplonchida, family Trichodoridae				
32	*Paratrichodorus porosus*	NC	Beijing Qinghewan	Creeping bentgrass	1
33	*Paratrichodorus minor*	EC	Suzhou Taihu, Suzhou Jinji Lake, Suzhou Shuangshan, Changzhou Kingswan, Nanjing Sun Island, Nantong Xiao Yang Kou, Shanghai Sino-Bay, Huangshan Pine	Creeping bentgrass, Seashore Paspalum, Hybrid bermudagrass	8
	Order Dorylaimida, family Longidoridae				-
34	*Xiphinema* spp.	EC	Huangshan Pine	Hybrid bermudagrass	1
		SC	Boao Asia BBS	Hybrid bermudagrass, Seashore Paspalum	1
35	*Xiphinema nuragicum*	EC	Suzhou Jinji Lake, Nanjing Sun Island, Nanjing Zhongshan, Shanghai Tomson	Creeping bentgrass	4
36	*Xiphinema hunaniense*	EC	Nanjing Sun Island	Creeping bentgrass	1
37	*Xiphinema insigne*	EC	Shanghai Sino-Bay	Creeping bentgrass	1
38	*Xiphinema ifacolum*	EC	Shanghai Sino-Bay, Nanjing Zhongshan	Creeping bentgrass	2
		SC	Shenzhen Sand River	Hybrid bermudagrass, Seashore Paspalum	1
39	*Paralongidorus koreanensis*	SC	Shenzhen Sand River	Hybrid bermudagrass	1
	Order Aphelenchida, family Aphelenchoididae				-
40	*Aphelenchoides pseudogoodeyi*	SC	Hainan Mission Hills, Boao Asia BBS	Hybrid bermudagrass, Seashore Paspalum	2
41	*Aphelenchoides bicaudatus*	EC	Shanghai Sino-Bay, Shanghai Tomson	Creeping bentgrass	2
		SC	Guangdong Chaoshan	Creeping bentgrass	1

NC: North China; EC: East China; SC: South China.

**Table 3:** The infestation, frequency, abundance, and damage threshold of the main nematode genera in golf courses.

Region ^1^	Genus ^2^	Infestation (%) ^3^	Frequency (%)	Abundance	Max (Individuals/100 cm^3^ of Soil)	Damage Threshold ^4^	Samples above Threshold (%) ^5^
NC	*Helicotylenchus*	80.00	75.00	1.89	215	600	-
	*Mesocriconema*	60.00	41.67	1.16	48	-	-
EC	*Filenchus*	35.71	21.15	1.65	392	-	-
	*Paratrichodorus*	64.29	59.62	1.36	103	80	9.68
	*Xiphinema*	42.86	23.08	1.86	328	200	8.33
	*Hoplolaimus*	35.71	23.08	1.47	135	150	-
	*Ditylenchus*	35.71	15.38	1.88	315	-	-
	*Meloidogyne*	21.43	13.46	1.61	103	100	28.57
SC	*Helicotylenchus*	83.33	78.26	2.55	2900	600	33.33
	*Hemicriconemoides*	66.67	47.83	1.05	25	-	-
	*Labrys*	33.33	17.39	1.20	45	-	-
	*Mesocriconema*	33.33	30.43	1.29	81	-	-
	*Meloidogyne*	16.67	34.78	1.37	105	100	12.5

^1^ NC: North China, EC: East China, SC: South China. ^2^ Nematode genera were selected according to the criteria of abundance greater than 1. ^3^ The infestation was defined as the number of golf courses having a particular nematode species divided by the total number of courses in this region, and is expressed as a percentage. ^4^ The damage threshold was defined as the number of nematodes required to cause economic damage on turfgrass, as suggested by Nelson (1995) and Stirling (2021). ^5^ Samples above threshold was defined as the percentage of samples above damage threshold in a specific region.

**Table 4:** Density and dominance of plant parasitic nematodes in healthy and problematic samples.

Golf Course	Health Status ^1^	Plant-Parasitic Nematodes Density (Individuals/100 cm^3^ of Soil)	Dominance (%) ^2^
Beijing Qinghewan	H	18	2.98
	P	33	2.78
Beijing Laffitte	H	57	5.55
	P	114	6.04
Beijing Yuyang	H	98	14.16
	P	89	17.87
Beijing CBD	H	217	27.78
	P	112	28.43
Suzhou Jinji Lake	H	19	2.64
	P	66	10.87
Suzhou Taihu	H	400	27.61
	P	322	28.10
Changzhou Kingswan	H	112	8.85
	P	67	6.73
Nanjing Sun Island	H	65	5.31
	P	333	41.73
Nanjing Zhongshan	H	241	24.69
	P	122	15.31
Shaanxi Yulin Desert	H	27	6.05
	P	91	18.80
Shanghai Sino-Bay	H	115	5.67
	P	127	12.10
Shanghai Links	H	137	10.83
	P	59	3.86
Shanghai Tomson	H	26	6.88
	P	127	31.91
Anhui Huangshan Pine	H	67	9.33
	P	32	4.18
Shenzhou Peninsula	H	82	14.86
	P	22	3.50
Wanning Shanqin Bay	H	2900	61.70
	P	423	50.12
Sanya Luhuitou	H	5	2.76
	P	19	10.86
Shenzhen Sand River	H	8	22.22
	P	108	65.85

^1^ H: healthy sample, P: problematic sample. Samples with symptoms of turfgrass damage (stunted growth, less dense turf stands, shoot chlorosis, and root discolor) were defined as problematic samples, while samples that did not show any symptoms were defined as healthy samples. ^2^ Dominance (%)is expressed as the percentage of the number of plant-parasitic nematodes in the total number of nematodes.

**Table 5:** Diversity of plant-parasitic nematodes in North, South, and East China.

Region	Number of Courses	Diversity *H’* ^1^	Richness *SR* ^2^	Evenness *J’* ^3^	Dominance *λ* ^4^	Diversity *H_2_* ^5^
NC	5	0.93 ± 0.01 b ^6^	60.98 ± 0.16 c	0.41 ± 0.04 b	0.62 ± 0.02 b	0.48 ± 0.02 b
EC	14	1.92 ± 0.01 a	89.56 ± 0.31 a	0.77 ± 0.05 a	0.17 ± 0.01 c	1.77 ± 0.05 a
SC	6	0.42 ± 0.02 c	88.36 ± 0.19 b	0.18 ± 0.03 c	0.85 ± 0.03 a	0.17 ± 0.03 c

^1^ Diversity *H’* = $-\overset{s}{\underset{i=1}{\sum }}pi\ln pi$, pi = proportion of individuals of taxa *i* in the total population. *H’* is commonly used to assess diversity, however, it may be dominated by the most abundant taxa or the overall number of taxa. ^2^ Richness *SR* = (*s* − 1) *ln N*, where *s* = the number of taxa (species) in the sample and *N* = the total number of nematodes identified in the sample. ^3^ Evenness *J’* = *H’*/*H’*_max_, where *H’*_max_ = *ln s.* ^4^ Dominance, λ = $\overset{s}{\underset{i=1}{\sum }}p_{i}^{2}$ is used to assess nematode dominance. ^5^ Diversity, *H_2_* = − *ln* *λ*, is an alternative measure of diversity. ^6^ Differences in the listed items were analyzed by Duncan’s multiple range test (*p* < 0.05). Different letters in a column in a group indicate significant differences between samples.

**Table 6:** Effect of turfgrass species on plant-parasitic nematode diversity in North, South, and East China ^1^.

Turfgrass Species	Number of Samples	Diversity *H’*	Richness *SR*	Evenness *J’*	Dominance *λ*	Diversity *H_2_*
Creeping bentgrass	57	2.13 ± 0.02 a ^2^	124.72 ± 2.15 a	0.77 ± 0.04 a	0.14 ± 0.02 c	1.97 ± 0.03 a
Seashore paspalum	17	0.21 ± 0.02 c	79.06 ± 0.97 b	0.09 ± 0.01 c	0.93 ± 0.02 a	0.07 ± 0.01 c
Hybrid bermudagrass	13	1.36 ± 0.03 b	42.89 ± 1.16 c	0.65 ± 0.04 b	0.33 ± 0.04 b	1.11 ± 0.05 b

^1^ The definitions of the indices in this table are as shown in Table 5. ^2^ Differences in the listed items were analyzed by Duncan’s multiple range test (*p* < 0.05). Different letters in a column in a group indicate significant differences between samples.

## Data Availability

Not applicable.
